# Seizure control in a patient with Dravet syndrome and cystic fibrosis^☆^

**DOI:** 10.1016/j.ebcr.2013.02.001

**Published:** 2013-03-29

**Authors:** Barbara Schmalbach, Bettina Moeller, Sarah von Spiczak, Hiltrud Muhle, Ulrich Stephani, Nicolas Lang

**Affiliations:** a Department of Neurology, Christian-Albrechts-University of Kiel, Arnold-Heller-Str. 3, Haus 41, 24105 Kiel, Germany; b Department of Neuropediatrics, Christian-Albrechts-University of Kiel, Arnold-Heller-Str. 3, Haus 9, 24105 Kiel, Germany

**Keywords:** DS, Dravet syndrome, SMEI, severe myoclonic epilepsy of infancy, *SCN1A*, neuronal voltage-gated sodium channel subunit type 1, CF, cystic fibrosis, TAP, Tests of Attentional Performance, TMT A, Trail Making Test, CFTR, cystic fibrosis transmembrane conductance regulator, GABA, γ-aminobutyric acid, Dravet syndrome, Bromide, Cystic fibrosis

## Abstract

Satisfactory treatment of patients with Dravet syndrome (DS) is often difficult. Some success can be achieved with bromides, but cognitive side effects and disturbed vigilance may limit their use. Here, we present the case of a successfully treated patient with DS and remarkable features in the course of his disease: additionally to DS, the patient was diagnosed with cystic fibrosis (CF), another genetic channelopathy. Seizure freedom could be achieved under treatment with potassium bromide at the age of 15, but at the age of 20, adverse events made it necessary to stop bromide treatment. After conversion to valproic acid, the patient remained seizure-free, and neuropsychological tests demonstrated sustained improvement of cognition.

## 1 Introduction

Dravet syndrome (DS, or severe myoclonic epilepsy of infancy (SMEI)) is characterized by temperature-sensitive and pharmacorefractory generalized seizures beginning in the first year of life, often evolving to status epilepticus. Some patients have additional complex partial seizures or atypical absence seizures. As the disease progresses, cognitive arrest or deterioration becomes evident in almost all patients in the second and third years of life after a normal initial psychomotor development. The long-term cognitive outcome in general is poor, most often with a low IQ, high mortality rate, and seizure persistence into adulthood [1]. Mutations of the neuronal voltage-gated sodium channel subunit type 1 gene (*SCN1A*) are the most frequent genetic causes of this syndrome [2]. Effective treatment is difficult [3], although barbiturates, benzodiazepines, valproic acid, and stiripentol were reported to be of some efficacy. Encouraging results have been demonstrated with bromides [4]; however, application is often limited by cognitive side effects and disturbed vigilance.

## 2 Case

### 2.1 Epilepsy and antiepileptic treatment

**Fig. 1:**
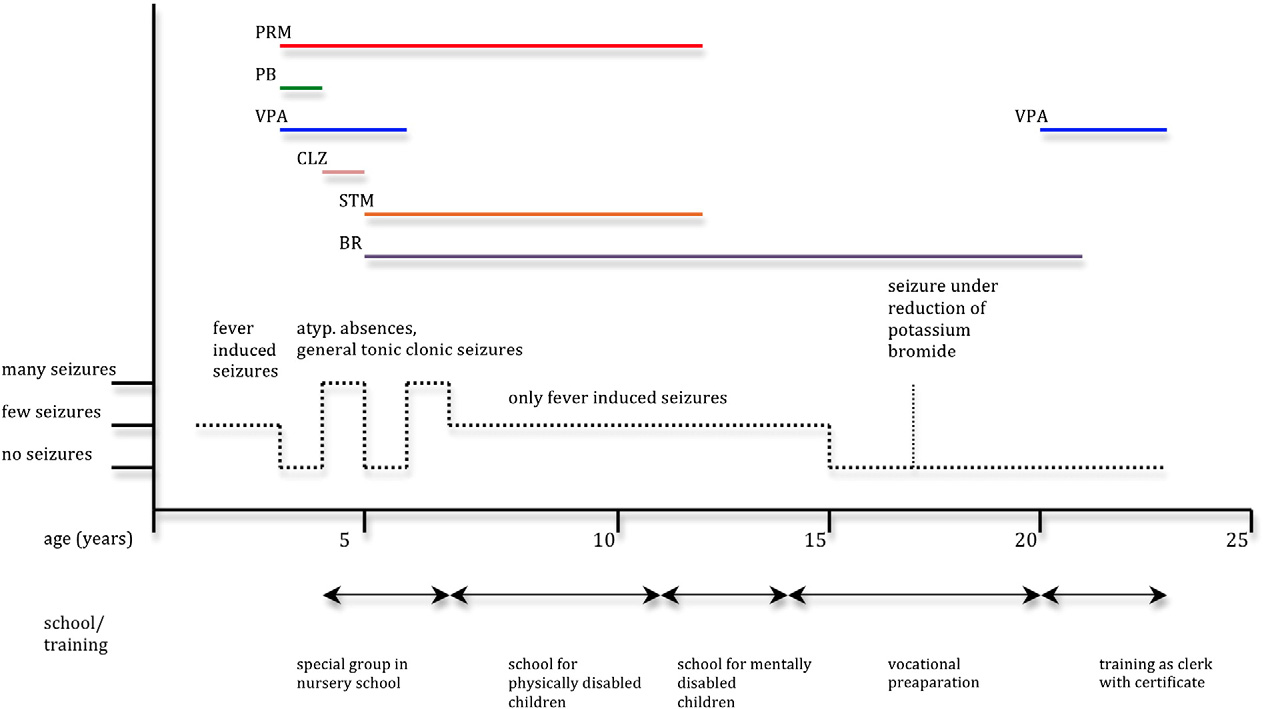
Schematic illustration of course of disease including medication, types and frequency of seizures, and education. When potassium bromide was reduced for the first time at the age of 17, a seizure occurred at a dosage of 850 mg/d, this is why the dosage of potassium bromide was subsequently increased up to 2125 mg. Under this treatment, the patient remained seizure-free once more. Due to intolerable side effects (cognition and skin), potassium bromide was tapered down again at the age of twenty, this time, over an interval of 8 months and after a treatment with valproic acid was initiated. The patient remained seizure-free under monotherapy with valproic acid.

We present the case of a male patient who developed, after diagnosis of cystic fibrosis (CF) at the age of three months, febrile seizures at the age of one and at the age of three, afebrile seizures, among them generalized tonic-clonic seizures, myoclonic seizures, and atypical absences. An MRI scan of the brain at the age of three did not reveal any abnormality. Given the clinical course, DS was suspected, and DNA sequence analysis of the *SCN1A* gene proved a de novo missense mutation, p.S1231T in exon 18, which results in an amino acid substitution in the D3S1 segment [5]. After insufficient sustained effect of antiepileptic treatment with benzodiazepines, barbiturates, primidone, sultiame, and valproic acid in varying combinations, the patient received potassium bromide in addition to valproic acid, primidone, and sultiame, which finally led to a cessation of seizures at the age of 7 years except for rare fever-induced seizures. Subsequently, valproic acid, primidone, and sultiame could be terminated, and the patient remained seizure-free under a monotherapy with 2550 mg of potassium bromide daily. Potassium bromide could be gradually reduced to 1700 mg without onset of seizures. At the age of 17, a further attempt of dose reduction was performed, and seizures reoccurred when the daily dose was reduced to 850 mg, but the patient became seizure-free once more with a daily dose of 2125 mg potassium bromide. At the age of 20, the patient was still seizure-free, but acne pustulosa and cognitive side effects were evident and attributed to the treatment with potassium bromide. A conversion of the antiepileptic therapy from potassium bromide to valproic acid was initiated, with an initial daily dose of 150 mg valproic acid being slowly increased weekly by 150 mg to a final daily dose of 1200 mg. Subsequently, bromide was slowly reduced over a period of eight months, with a reduction of 425 mg every two months. Since then, the patient remained seizure-free under a monotherapy with valproic acid for more than two years, and he is presently 23 years old (Fig. 1).

### 2.2 Cognitive development

**Fig. 2:**
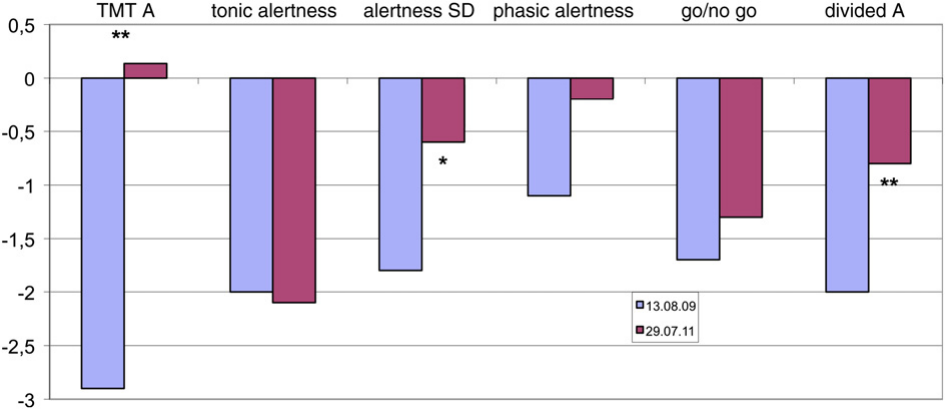
Comparison of neuropsychological assessment during (2009) and after (2011) therapy with potassium bromide. The figure shows z-values (m = 0, SD = 1), i.e., − 1 < x < 1; 1 is average, < − 1 is below average, and < − 2 is far below average. *Difference > 1 SD, **Difference > critical difference.

Psychomotor development gradually deteriorated in the first year after initial status had been normal. At the age of three, the patient went to a regular nursery school with a special group for mentally handicapped children followed by diverse supportive schools for physically and mentally disabled children and subsequent vocational preparation. After discontinuation of primidone, cognition improved for a short period; subsequently, the patient was able to learn to read. Nevertheless, due to the therapy with potassium bromide, cognitive abilities continued to be impaired. Neuropsychological assessment during ongoing bromide therapy included IQ screening, cognitive speed of processing, attention, verbal, and nonverbal memory, verbal and nonverbal fluency, construction and orientation, and the test batteries for the evaluation of attention derived from the Tests of Attentional Performance (TAP). The testing revealed deficits in cognitive speed of processing (Trail Making Test, TMT A), tonic alertness, phasic alertness, divided attention, and selective attention and verbal fluency with an estimated IQ of 90. A second neuropsychological assessment after discontinuation of bromide therapy and conversion to valproic acid confirmed the clinical impression of a clinically relevant improvement of alertness, divided attention, and cognitive speed of processing (TMT A) (Fig. 2).

At the age of 23 years, he successfully finished his training as a clerk, which he had begun 3 years ago.

### 2.3 Cystic fibrosis

The patient we report of was diagnosed with cystic fibrosis (CF) in his first year of life, after a homozygous mutation in the cystic fibrosis transmembrane conductance regulator (*CFTR*) gene (p.F508 del) was found. In the following years, the course of CF was rather uncomplicated. Although an exocrine and endocrine pancreatic insufficiency emerged, pulmonary infections were rare.

## 3 Discussion

Classic clinical features of DS characterize our patient's course of disease. However, the following two important features make this case different from other cases:

1. It is remarkable that the patient was not only diagnosed with DS, but also with CF, both genetic channelopathies confirmed by sequence analysis. No concomitant existence of these two genetic diseases has been described in the literature so far. Yet, a combined inheritance is difficult to postulate since the *CFTR* gene is located on chromosome 7q31.2 [6], whereas the *SCN1A* gene is located within a cluster of three sodium channel genes, including *SCN2A* and *SCN3A*, on chromosome 2q24 [7]. Furthermore, the *CFTR* mutation is inherited from the patient's parents by an autosomal recessive inheritance, whereas the *SCN1A* mutation is a de novo mutation in our patient with a negative parental testing. Of the cases with DS, 70–80% is caused by mutations of the *SCN1A* gene, 90% of which occur de novo [8]. Genetic modifiers [9] and environmental factors probably contribute to the variable phenotype of patients with *SCN1A* mutations.
2. The other remarkable fact is the relatively benign course of both diseases. Intractable seizures are most commonly a hallmark of DS. Pharmacological treatment is difficult and ineffective in many patients [3]. A favorable effect of bromides has been recently reported [4] whose benefit as antiepileptic pharmacotherapy was first described in 1853. Because of side effects involving skin, gastrointestinal tract, and the nervous system, bromides were, for a long time, only considered as an adjunctive therapy for children and adolescents whose generalized tonic-clonic seizures did not respond to conventional antiepileptic drugs [10]. The real mode of action has not yet been elucidated, though some studies reported that the antiepileptic mode of action was through γ-aminobutyric acid (GABA), and bromide might potentiate the effect of GABA by hyperpolarizing the postsynaptic membrane [11].

In our patient, potassium bromide was highly effective but led to severe skin problems that prompted us to discontinue bromide therapy at the age of twenty and to change back to valproic acid, which had been of limited efficacy before. Subsequently, cognitive function, i.e., divided attention, alertness, and speed of cognitive processing, improved. The observation of only moderate mental disability in adulthood stands in contrast to the previously held notion that DS had a relatively severe cognitive prognosis.

Whether the mild course of both diseases, DS and CF, is due to the coexistence of both channelopathies is difficult to say. An interaction on a genetic basis is rather unlikely as illustrated above. However, there might be an explanation on a functional level: Epithelial sodium channels are expressed in alveolar cells and play a crucial role in ion and fluid regulation of the lung. Hyperactivity of these epithelial sodium channels due to loss of CFTR-mediated inhibition in patients with CF leads to some of the symptoms present in cystic fibrosis due to hyperabsorption of sodium resulting in airway dehydration [12]. Having this in mind, an influence of the mutation in the CFTR gene on channels other than epithelial sodium channels (for example *SCN1A*-encoded sodium channels) might be hypothesized which could then lead to an amelioration of DS-derived symptoms.

## 4 Summary

This case report emphasizes the importance of ongoing rational therapy changes when seizures in patients with epilepsy are treated unsatisfactorily. Significant benefit can be achieved even after years of drug resistance and when numerous attempts to reach seizure freedom had failed before. Certainly, this case has many circumstances, such as the unusual pathogenesis with two distinct channelopathies, that make transferability to other epilepsy syndromes difficult. Still, it demonstrates the significance of reduction or discontinuation of medication with severe impact on cognitive function.

## Diclosures

The authors have nothing to disclose.

## Conflict of interest

The authors declare no conflicts of interest.
